# Injury patterns, management and outcomes of hemodynamically unstable pelvic fractures at an Australian level 1 trauma centre: a 10-year registry analysis

**DOI:** 10.1007/s00068-026-03288-0

**Published:** 2026-08-24

**Authors:** Luan Louw, Kyle Raubenheimer, William G. Blakeney, Samuel Young, Zsolt J. Balogh, Dieter G. Weber

**Affiliations:** 1 Trauma Service, Royal Perth Hospital, Perth, Australia; 2 Department of Orthopaedic Surgery, Royal Perth Hospital, Perth, Australia; 3 Department of Traumatology, John Hunter Hospital, Newcastle, Australia; 4 University of Newcastle, Callaghan, Australia; 5 University of Western Australia, Crawley, Australia

**Keywords:** Pelvic fracture, Pelvic ring injury, Haemodynamic instability, Traumatic shock, Trauma system

## Abstract

**Introduction/background:**

Pelvic ring injuries (PRI) in traumatic shock are associated with over 30% mortality even in high income countries’ mature trauma systems, but recent Australian data showed no haemorrhage related mortality in this group. We aimed to describe the epidemiology, management and outcomes of PRI with haemodynamic instability managed in a state’s only Level 1 adult trauma centre.

**Design:**

We conducted a retrospective cohort study of all trauma patients with major PRI (Abbreviated Injury Scale (AIS) > 3) and hemodynamic instability (defined as systolic blood pressure (SBP) <90 mmHg or a shock index (heart rate (HR)/SBP) > 0.7) presenting to the state adult trauma centre between 2013 and 2022. Data on demographics, injury patterns, management strategies, and outcomes were collected from the State Trauma Registry and hospital records.

**Results:**

Of 1369 patients with pelvic or acetabular fractures during the 10-year study period, 152 (11%) had major PRI with hemodynamic instability at presentation. Most were male (72%) with a median age of 35 years. Motor vehicle and motorbike crashes accounted for 52% of injuries. The median Injury Severity Score was 33. Massive transfusion protocols were activated in 45% of cases, while angioembolisation was utilised in 5.9%. Overall in-hospital mortality was 7.2% and mortality due to haemorrhage was 2%.

**Conclusions:**

In our mature trauma system, hemodynamically unstable PRI patients’ low mortality is consistent with the recently reported outcomes of best performing centres.

Level of Evidence

## Introduction

Pelvic ring injuries (PRI) caused by high-energy trauma may result in serious injuries associated with considerable morbidity and mortality [1, 2]. Haemorrhage associated with PRI is well recognised, and a critical component in patients that present with traumatic shock [3]. Management strategies of bleeding in patients with hemodynamically unstable PRI includes angioembolisation, resuscitative endovascular balloon occlusion of the aorta (REBOA), preperitoneal pelvic packing (PPP) and pelvic external fixation [5–7]. Historically Australian and international studies have shown significant mortality rates for pelvic fractures ranging from 8% to 37% [1–9, 11]. Current data shows that even high-income countries still have high mortality rates [3, 4]. A recent prospective study of multiple level 1 trauma centres in the US reported a mortality rate of 32% in patients presenting with PRI and shock [3]. Given the considerable mortality rates, systematic efforts have been made within Australia over the last 20 years to improve outcomes. These include protocolised prehospital use of pelvic binders and haemostatic resuscitation (initiation of tranexamic acid and blood products including PRBC and FFP). Furthermore, in hospital there have been advancements in targeted medical resuscitation (including use of point of care blood gas, thromboelastography and major haemorrhage protocol), improved access to angioembolisation and early definitive fixation [8]. With these implemented changes, more recent Australian literature has shown improved mortality rates and even recently no haemorrhage related mortality from PRI [8]. The decision for the optimal management strategy remains complex, and includes factors such the degree of hemodynamic instability, quality of resuscitation, imaging modalities and centre resources [10, 11]. The primary aim of this project is to describe the trend in the annual number of major trauma patients with major PRI with haemodynamic instability presenting to a major trauma centre over a 10-year period. Secondary outcomes included a description of the Western Australian cohort cohort in terms of clinical outcomes, resource utilisation and a comparison between those patients who survived and those who died to determine risk factors associated with death.

## Methods

### Study design

This was a retrospective cohort conducted over a 10-year period between 01 January 2013 and 31 December 2022 using data from the Western Australian State Trauma Registry, transfusion medicine service records at Royal Perth Hospital and focused review of the digital medical records. This study was reported in accordance with the Strengthening the Reporting of Observational studies in Epidemiology (STROBE) guidelines for observational trials using routinely collected clinical data. Approval was sought and obtained from the Clinical Safety and Quality Unit in East Metropolitan Health Service (GEKO QA: 50626) prior to commencing the study.

### Setting

Royal Perth Hospital is the only level 1 trauma centre within Western Australia. Western Australia spans 32.7% of the Australian continent with an area of 2 527 013 square kilometres [12]. Royal Perth Hospital receives the second largest amount of major trauma patients in Australia, servicing a population of 2.43 million and 2.76 million people in 2012 and 2021 respectively [13, 14], totalling around 1100 patients annually.

The State Trauma Unit is comprised of trauma consultants (consultant general surgeons who have undertaken a trauma fellowship), trauma fellows (post-fellowship trainees completing a trauma fellowship), non-specialist training resident medical staff, as well as dedicated nursing and allied health staff subspecialised in trauma care. The unit works closely and in collaboration with the pre-hospital services and the emergency department for optimal triage and management of the injured patients. Royal Perth Hospital has access to around the clock interventional radiology and endovascular suites, as well as orthopaedic surgeons with pelvic subspecialisation.

At our institution, initial management of suspected pelvic haemorrhage consists of pelvic stabilization with a binder, concurrent haemostatic resuscitation with early activation of the major haemorrhage protocol when triggers are met, and rapid assessment for competing sources of shock. Where physiology permits, early CT is used to define injury pattern and identify arterial extravasation to guide interventional radiology. Patients with ongoing hemodynamic compromise despite resuscitation are managed with expedited haemorrhage control according to physiology and suspected bleeding source, including angioembolization for suspected pelvic arterial bleeding, operative pelvic stabilization with or without adjunctive preperitoneal pelvic packing, and selective use of REBOA in extremis as a bridge to definitive haemorrhage control. Management decisions are made collaboratively by trauma team, emergency medicine, orthopaedic surgery and interventional radiology teams in real time.

### Participants

All major trauma patients presenting to Royal Perth Hospital with a PRI were screened for eligibility. Major trauma was defined as an Injury Severity Score (ISS) of greater than 12. All patients with major PRI, defined as an Abbreviated Injury Scale (AIS) of greater than 3 and with haemodynamic instability on presentation were included. Royal Perth Hospital accepts trauma patients aged 14 years and older. Patients were defined as having hemodynamically instability if their systolic blood pressure (SBP) was less than 90 mmHg or a shock index (heart rate (HR)/SBP) was greater than 0.7. The shock index threshold of 0.7 was used as it has shown to have an acceptable balance between sensitivity and specificity when predicting the need for haemostasis intervention in an emergency department population [15]. There were no exclusion criteria.

### Variables: patient demographics

Patient demographics including age, sex, and ethnicity were collected for this study.

### Variables: injury details

Details surrounding the injury were collected. These included mechanism of injury, date of injury, and whether injury occurred in the rural or metropolitan area. The severity of injury was described by the ISS, and the injury pattern is by the individual body regions’ AIS.

### Variables: initial presentation

Details on the initial presentation were collected. These included logistical variables (time of arrival, mode of arrival, and place where the patient was transferred from), initial vital signs (HR, SBP), and initial biochemical variables (pH, base excess, lactate, haemoglobin (Hb), international normalised ratio (INR), fibrinogen).

### Variables: management

Management was split into four pragmatic categories: emergency management, resuscitative management in the first 24 h since injury, definitive haemorrhage control, and definitive pelvic fixation. Emergency management variables included activation of the major transfusion protocol (MTP), use of a pelvic binder, and/or use of a resuscitative endovascular balloon of the aorta (REBOA). Resuscitative management in the first 24 h included the number of units of blood products used (packed red blood cells (PRBC), fresh frozen plasma (FFP), platelets, or cryoprecipitate), and use of tranexamic acid (TXA). For definitive haemorrhage control, time and type of definitive haemorrhage control were collected. Time and type of definitive pelvic fixation were collected using individual case files. Time and type of definitive pelvic fixation were collected using individual case files. Time periods were captured in the registry as: within 24 h, within 1 week, and after 1 week.

### Variables: outcomes

The primary outcome of this project was the annual number of pelvic fractures with haemodynamic instability in major trauma patients as prospectively collected through the trauma registry. Secondary outcomes of clinical outcomes and resource utilisation related outcomes were collected for patients’ hospital stay. These included emergency department (ED) length of stay (LOS), ED dispatch destination, hospital LOS, intensive care unit (ICU) LOS, final discharge destination, in-hospital mortality, and cause of in-hospital mortality.

### Statistical analysis

Descriptive statistics were calculated for all demographic variables. All data was tested for normality using Q-Q plots and histogram analysis. For parametric data, means and standard deviation were used to describe centre values. For non-parametric data, median with interquartile range (IQR) was used for descriptive statistics. For the primary outcome (annual number of major PRI with haemodynamic instability), we utilised a Poisson regression analysis to determine if a statistically significant trend was present. To determine risk factors for mortality, comparisons between patients who survived and patients who died were conducted. For categorical variables, chi-squared tests or Fisher Exact tests were used when sample sizes were less than 5. For continuous variables, students t-tests or Wilcoxon-signed rank tests were used as determined by normality. Statistical significance was set at *p* < 0.05. All statistical analysis and data visualisation was performed using R Statistical Software [16].

## Results

**Fig. 1:**
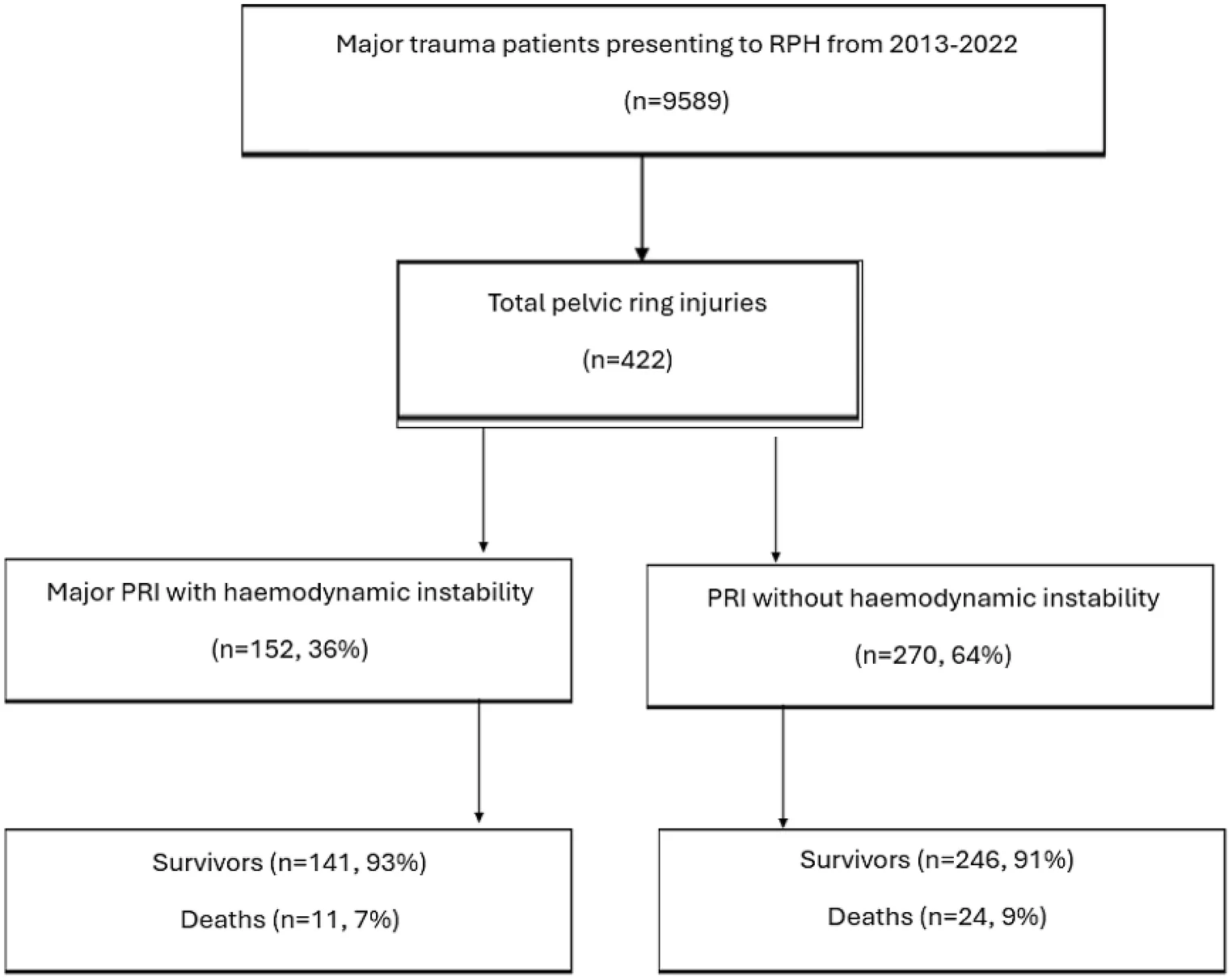
Flow diagram of inclusion and exclusion criteria for patient population. RPH – Royal Perth Hospital; PRI – pelvic ring injury; AIS – abbreviated injury scale; SBP – systolic blood pressure; HR – heart rate

**Fig. 2:**
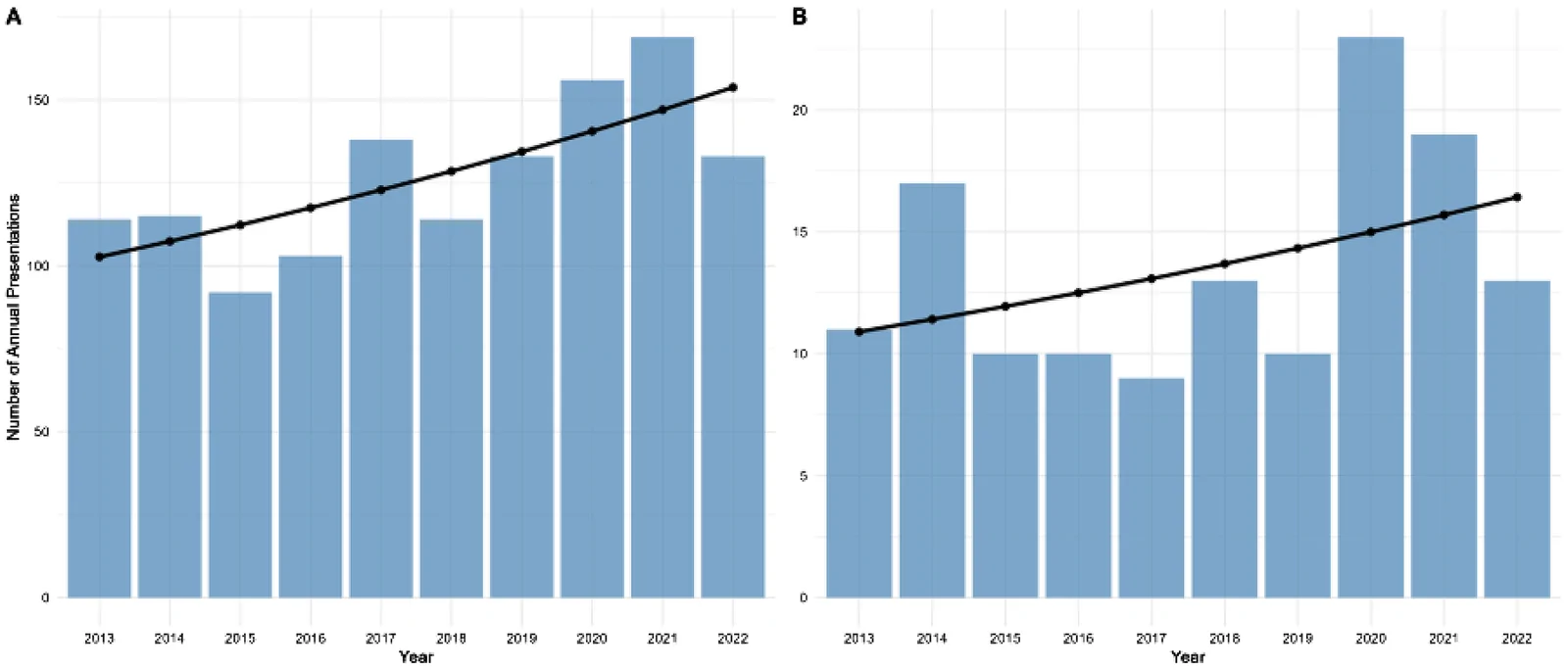
Annual presentations of patients with major pelvic ring injuries with hemodynamic instability between 2013 to 2022 in Royal Perth Hospital. Graph **A**: Number of annual pelvic ring injuries presenting to Royal Perth Hospital between 2012 to 2022. Graph **B**: Patients with haemodynamic instability and pelvic ring injuries

Between 2013 and 2022, a total of 9589 major trauma patients (defined as ISS > 12) presented to RPH. Of these, 422 had PRI and 152 (36%) patients had major PRI coupled with haemodynamic instability (Fig. 1). The annual number of patients with PRI presenting to RPH during the period increased by 4.6% (*p* < 0.001; 95% CI: 1.02–1.08). The annual number of major trauma patients with PRI and haemodynamic instability did not change significantly over the 10-year period (*p* = 0.13; 95% CI: 0.98–1.11; Fig. 2). Demographics for all patients are detailed in Table 1. Most patients were male (72%), median age of 35 years (IQR 24;53), and from the metropolitan area (71%). The three most common mechanisms of injury were motor vehicle crashes (27%), motorbike crashes (25%), and falls (17%). The median ISS was 33 (IQR: 24–41).

**Table 1:** Demographics and injury patterns of Western Australian patients with a severe pelvic ring injury and hemodynamic instability between 2013 and 2022

Variable	Total (*n* = 152)	Survivors (*n* = 141)	Deaths (*n* = 11)	*p* value
Age	35 (24, 53)	34 (24, 50)	47 (35, 65)	
Sex				
Male	109 (72%)	102 (72%)	8 (73%)	0.95
Female	43 (28%)	40 (28%)	3 (27%)	
Ethnicity				
Aboriginal or TSI	10 (6.5%)	10 (7%)	0	NA
Other	141 (3%)	131 (93%)	10 (91%)	
Unknown	1 (< 1%)	1 (< 1%)	0	
Location of Injury			-	
Metropolitan	108 (71%)	97 (69%)	11 (100%)	0.03
Rural	44 (29%)	44 (31%)	0	
ISS	33 (24, 42)	30 (24, 41)	59 (47, 66)	
AIS Head and Neck ≥ 3	36 (24%)	28 (20%)	8 (73%)	0.34
AIS Thorax ≥ 3	70 (46%)	60 (42%)	10 (91%)	
AIS Abdomen ≥ 3	54 (35%)	48 (34%)	6 (55%)	

TSI – Torres Strait Islander; MBC – motorbike crash; MVC motor vehicle crash; ISS – injury severity score; AIS – abbreviated injury scale

The median pelvic AIS was 4 (IQR: 4–5) in survivors and 5 (IQR: 4–5) in non-survivors. On arrival, there was no significant difference between survival and death cohorts for HR, SI, Hb, WCC, neutrophils, INR, fibrinogen, initial pH, or initial lactate (Table 2). Systolic blood pressure was higher in patients who died as compared to those who survived (Table 2).There were significant differences in platelets, lymphocytes, initial BE, worst pH, worst BE and worst lactate (Table 2) between patients who died and patients who survived.

**Table 2:** Comparison between survivors and deaths of initial clinical and biochemical results of Western Australian patients with a severe pelvic ring injury and hemodynamic instability between 2013 and 2022

Initial Presentation to ED	Total (*n* = 152)	Survivors (*n* = 141)	Deaths (*n* = 11)	*p* value
HR	96 (80, 111)	95 (80, 110)	106 (93, 147)	*p* = 0.11
SBP	116 (100, 135)	115 (100, 133)	141 (115, 147)	***p*** < 0.05*
SI	0.81 (0.63, 1.0)	0.81 (0.63, 1.0)	0.8 (0.71, 0.91)	*p* = 0.85
Hb	131 (121, 140)	132 (121, 141)	121 (104, 128)	*p* = 0.06
WCC	18.7 (14, 23.5)	18.7 (14.1, 23.8)	18.7 (14.3, 22)	*p* = 0.82
Platelets	248 (202, 29)	249 (204, 297)	204 (195, 234)	***p*** < 0.01**
Neutrophils	15 (11.6, 19)	15.1 (11.6, 19.3)	13.4 (10.5, 17.4)	*p* = 0.47
Lymphocytes	2.1 (1.4, 3.3)	2.1 (1.4, 3.2)	3.3 (2.9, 3.9)	***p*** < 0.05*
INR	1.1 (1.1, 1.3)	1.1 (1.1, 1.3)	1.3 (1.3, 1.5)	*p* = 0.06
Fibrinogen	2.5 (1.9, 2.9)	2.5 (1.9, 2.9)	1.9 (1.6, 2.5)	0.23
Initial pH	7.3 (7.2, 7.3)	7.3 (7.2, 7.3)	7.2 (7, 7.3)	0.12
Initial BE	-4 (-7, -2)	-3 (-6, -1)	-8 (-15, -4)	***p*** < 0.05*
Initial Lactate	3.1 (2.3, 4.3)	2.9 (2.1, 4)	4 (3.3, 8.7)	0.07
Worst pH within 6 h	7.3 (7.2, 7.3)	7.3 (7.2, 7.3)	7.1 (7, 7.2)	***p*** < 0.01**
Worst BE within 6 h	-6 (-8, -4)	-5 (-7, -4)	-12 (-17, -7)	***p*** < 0.01**
Worst Lactate within 6 h	2.8 (1.8, 4.1)	2.6 (1.6, 3.8)	6.4 (3.7, 9.9)	***p*** < 0.01**

ED - Emergency Department; HR – heart rate; SBP – systolic blood pressure; SI – shock index; Hb – haemoglobin; WCC – white cell count; INR – international normalized ratio; BE – base excess; hrs - hours

Sixty-nine (45%) patients received blood products. There was a significant difference in the median number of packed red blood cells between cohorts, with patients who survived receiving four units (IQR: 2–5 units) whilst patients who died received 25 units (IQR: 10–31 units). For those patients who received FFP and platelets, there was a significant difference between the two groups. There was a significant difference in the use of a major haemorrhage protocol between the two cohorts (31% vs. 82% of patients who died).

Management of hemodynamically unstable patients is detailed in Table 3. Most patients had documented use of a pelvic binder, with no difference between cohorts. Nine patients (5.9%) received angioembolisation with a median (Q1-Q3) time to from arrival to emergency department to interventional radiology of 129 (IQR: 81,- 184) minutes. Two patients were managed using REBOA and two with PPP. Over two-thirds (*n* = 105, 69%) of patients received some form of pelvic operation, with the median (Q1-Q3) time to fixation being 63 (27–108) hours from presentation (Fig. 2). Majority of these were open reduction and internal fixation.

**Table 3:** Comparison between survivors and deaths of ED management and blood product usage of Western Australian patients with a severe pelvic ring injury and hemodynamic instability between 2013 and 2022

ED Management	Total (*n* = 152)	Survivors (*n* = 141)	Deaths (*n* = 11)	*p* value
PRBC Transfusion	69 (45%)	60 (43%)	9 (82%)	***p*** < 0.05*
* Median PRBC Amount Transfused*	4 (2, 8)	4 (2, 5)	25 (10, 31)	***p*** < 0.01**
Number of Patients Receiving FFP	62 (41%)	54 (38%)	8 (73%)	
FFP Median	2 (2, 5)	2 (2, 4)	21 (6, 25)	***p*** < 0.01**
Number of Patients Receiving Platelets	35 (23%)	26 (18%)	9 (82%)	
Platelets Median	1 (1, 6)	1 (1, 5)	5 (1, 6)	***p*** < 0.01**
MHP Use	53 (35%)	44 (31%)	9 (82%)	***p*** < 0.01**
Pelvic Binder	125 (82%)	114 (80%)	11 (100%)	*p* = 0.21
REBOA	4 (2.6%)	2 (1.4%)	2 (18%)	*P* = 0.027
TXA	64 (42%)	56 (39%)	8 (73%)	*p* = 0.054

ED - Emergency Department; PRBC – packed red blood cells; FFP – fresh frozen plasma; MHP – major haemorrhage pack; REBOA – resuscitative endovascular balloon occlusion of the aorta; TXA – tranexamic acid

With regards to disposition, patients spent a median of nearly 2 h and 27 min in the emergency department, with no difference between groups (*p* = 0.59). Hospital length of stay was significantly longer for patients who survived as compared to those who died (17 days vs. 2 days, *p* < 0.01). For patients who survived, most were discharged to rehabilitation (*n* = 93, 61%), followed by discharge home (*n* = 29, 19%), and then transferred to other hospitals (*n* = 15, 10%). Two patients discharged against medical advice, and 2 patients were transferred to residential care facilities. Eleven patients (7.2%) succumbed to their injuries. The most common cause of death was head injury followed by haemorrhage. Non-pelvic operative procedures were performed in 8 of the 11 non-survivors (72.7%), including laparotomy in 5 patients (45.5%), craniotomy or neurosurgical intervention in 2 patients (18.2%), and thoracotomy in 1 patient (9.1%). Three patients (2%) died due to haemorrhage. These patients had severe traumatic shock on presentation with initial BE < 14 and pH < 7.05. Of the fatalities, 2 patients were managed with REBOA, had pelvic angiography without embolization and underwent early surgical pelvic stabilisation. The third patient underwent damage control surgery with trauma and vascular surgery for management of traumatic aortic and iliac artery injury. One patient died due to multiple organ failure secondary to haemorrhage. This patient underwent angioembolisation with subsequent cardiac arrest in interventional radiology followed by trauma laparotomy and PPP. The mortality among the haemodynamically normal patients was higher, however this group was older (39 vs. 35 years) and had higher rates of major head injury (AIS ≥ 3: 36.6% vs. 24%) and chest trauma (AIS ≥ 3: 75% vs. 46%), with proportionally more deaths attributable to traumatic brain injury, respiratory complications, and cardiac failure.

## Discussion

The primary aim of our project was to determine the annual incidence of patients with major PRI and haemodynamic instability presenting to the only level 1 trauma centre in Western Australia. The number of major trauma patients presenting to the only level 1 trauma centre is stable over the time period 2013 to 2022. The annual incidence of major trauma patients with a major PRI increased over the period 2013 to 2022 (*p* < 001). Interestingly, annual incidence of major PRI with haemodynamic instability presenting to the same centre did not increase (*p* = 0.13). The reasons for this discrepancy are unclear, however may be due to the increased availability of advanced imaging modalities allowing for detection of less severe injuries which may otherwise have been missed. Secondly, maturation of the state-trauma system may have led to increased numbers of low- to moderate severity injuries being transferred to the centre whilst presentations of severe injuries to the level 1 trauma centre would have remained constant. Finally, the population of Perth increased by over 200 000 people over the study period [13, 14]. Increased numbers of less severe injuries may have been influenced by the growing population of Western Australia. These factors may help explain why there was an increase in PRI but no increase in patients with major PRI and haemodynamically instability. It should be noted that our data does not represent a true Western Australian incidence of major PRI with haemodynamic instability. Incidence rates of PRI have previously been reported to range from 8 to 23 fractures per 100,000 person-years [19, 20]. Patients with low-energy trauma or minor pelvic fractures may have been managed at other hospitals, and high-energy injuries resulting in death at the scene would not have been included. Therefore, this project cannot provide an accurate estimate of PRI incidence across Western Australia [13, 14]. Comprehensive epidemiological studies combining multiple data sources, including regional hospitals and prehospital data, are needed to determine the true population-level incidence of PRI.

Of the 422 patients presenting with PRI, 152 (36%) had major PRI with haemodynamic instability, consistent with the reported prevalence of physiologically compromised pelvic fracture patients in other major trauma systems [2, 4, 9–11]. The demographic profile aligns with published experiences elsewhere, showing the predominance of young to middle-aged males and in the population with primarily high-energy mechanisms [1–4, 9–11]. The systolic blood pressure was higher in non survivors. There may be two reasons for this. In our cohort those dying from traumatic brain injury had a higher median ED SBP (148 mmHg, IQR 110–149) compared to those dying from other causes (120.5 mmHg, IQR 97–143), consistent with a hypertensive response to intracranial pathology. The older non-survivor patients also likely contributed to higher SBP due to more frequent chronic disease and pre-existing hypertension, independent of acute haemodynamic status.

The in-hospital mortality in this study (7%) is comparable to the lowest mortality rates reported nationally and internationally [7, 10]. Previously mortality rates for pelvic fractures have been reported to be up to 37% [1–9, 11]. Two recent American studies examined mortality in PRI with traumatic shock, reporting mortality rates of 32% and 36% in patients presenting with both shock and PRI [3, 4]. Although our cohort had a higher median ISS (33 vs. 28 and 24), the American cohorts presented with more severe physiological derangement (heart rate 96 vs. 107 and 116; systolic blood pressure 116 vs. 101 and 91). Differences in the definition of haemodynamic instability may account for some of this variation. Our study and that by Costantini et al. defined instability based on markers of shock at presentation, whereas Anand et al. used transfusion requirements and pelvic haemorrhage control interventions as criteria [3, 4]. Despite these discrepancies, the reason for the lower mortality observed in our cohort remains unclear. Consistent with our findings, a recent Australian study reported no haemorrhage-related deaths over a 10-year period [8]. The authors attributed these outcomes to immediate access to blood products, a mature resuscitation protocol, and rapid availability of angioembolisation and surgical intervention. Our centre has also previously described the effectiveness of angioembolisation for arterial haemorrhage related to PRI. Over a 15-year period the mortality rate of patients who underwent angioembolisation was 10% [21]. Given our low mortality rate due to haemorrhage of 2%, this suggests that despite the Western Australian geographic challenges, reasonable outcomes can be achieved.

**Fig. 3:**
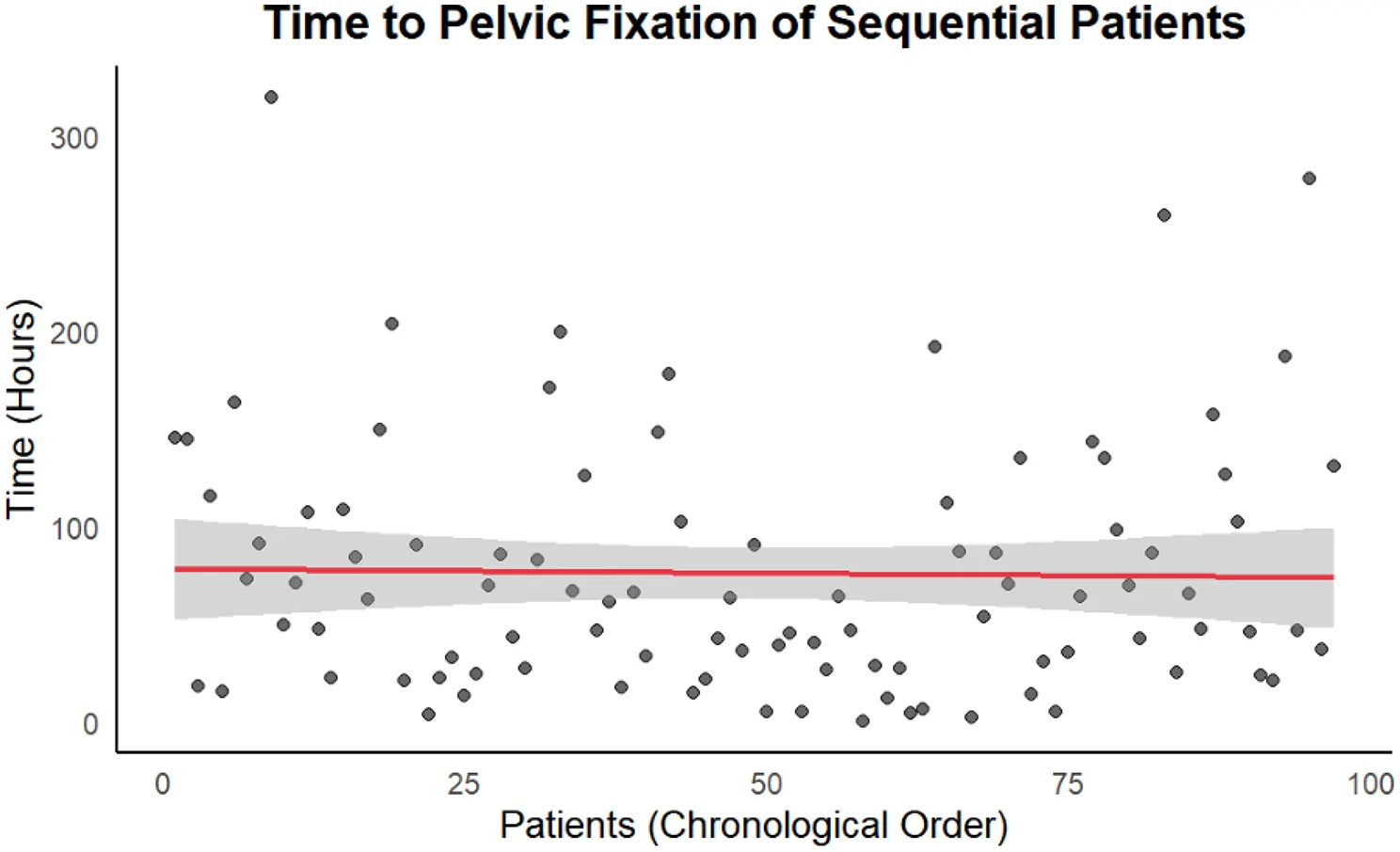
Time to pelvic surgical fixation of sequential patients between 2013 and 2022

Our cohort demonstrated high utilisation of massive transfusion protocols, with non-survivors receiving a median of 25 units of packed red blood cells compared to 4 units in survivors, underscoring the severity of haemorrhage and coagulopathy in this group. This is consistent with international data linking transfusion volume to mortality in pelvic trauma [17, 18]. Early application of pelvic binders was achieved in most patients (Table 3), whereas definitive haemorrhage control via angioembolisation was infrequent (5.9%). This low utilisation may reflect the predominance of venous bleeding, rapid stabilization through resuscitation, or patient factors precluding endovascular intervention. Our time to angioembolisation is also comparable to other centres. Agolini et al. found a significantly greater survival rate in patients embolised with 3 h of presentation, however Tanizaki et al. found lower mortality rates for patients embolised within 60 min [22, 23]. In our cohort, there were two mortalities in patients who were embolised, one patient received angioembolisation at 6 h and the other at 81 min. Surgical fixation was performed in 69% of patients. The median time to fixation was 63 h and this time did not seem to change during the study period, suggesting potential opportunities to optimise the timing and coordination of definitive management (Fig. 3).

We acknowledge several limitations in the study. Firstly, this was a retrospective study using registry data from a single level 1 trauma centre which has potential for selection bias, and temporal bias over the 10-year period. Secondly this project did not report on prehospital deaths or patients who were managed peripherally and not transferred to the Level-1 Trauma Centre. Although pelvic binder application is protocol-driven for suspected pelvic injuries in the prehospital setting, differentiation between prehospital and in-hospital binder use was not possible in this dataset. Similarly, whilst in-hospital blood product administration was recorded, transfusion data for patients transferred from another centre was not recorded as a distinct variable. Therefore, some patients may have received blood products prior to arrival that is not accounted for in this analysis. Finally, there are limitations from our chosen definition of haemodynamic instability using SBP and shock index.

## Conclusion

In our mature trauma system, the number of hemodynamically unstable PRI patients did not significantly change over a 10-year period. The overall mortality rates were low, with limited need for angioembolisation.

## Data Availability

The data that support the findings of this study are not publicly available due to ethical and privacy restrictions related to patient confidentiality and registry governance. De-identified data may be available from the corresponding author upon request and with approval from the relevant data custodians.
